# Antiviral Peptide-Based Conjugates: State of the Art and Future Perspectives

**DOI:** 10.3390/pharmaceutics15020357

**Published:** 2023-01-20

**Authors:** Toni Todorovski, Daniela Kalafatovic, David Andreu

**Affiliations:** 1 Department of Medicine and Life Sciences, Universitat Pompeu Fabra, 08003 Barcelona, Spain; 2 University of Rijeka, Department of Biotechnology, 51000 Rijeka, Croatia

**Keywords:** peptide-drug conjugates, antivirals, microbial infections

## Abstract

Infectious diseases caused by microbial pathogens (bacteria, virus, fungi, parasites) claim millions of deaths per year worldwide and have become a serious challenge to global human health in our century. Viral infections are particularly notable in this regard, not only because humankind is facing some of the deadliest viral pandemics in recent history, but also because the arsenal of drugs to combat the high levels of mutation, and hence the antigenic variability of (mostly RNA) viruses, is disturbingly scarce. Therefore, the search for new antivirals able to successfully fight infection with minimal or no adverse effects on the host is a pressing task. Traditionally, antiviral therapies have relied on relatively small-sized drugs acting as proteases, polymerases, integrase inhibitors, etc. In recent decades, novel approaches involving targeted delivery such as that achieved by peptide–drug conjugates (PDCs) have gained attention as alternative (pro)drugs for tackling viral diseases. Antiviral PDC therapeutics typically involve one or more small drug molecules conjugated to a cell-penetrating peptide (CPP) carrier either directly or through a linker. Such integration of two bioactive elements into a single molecular entity is primarily aimed at achieving improved bioavailability in conditions where conventional drugs are challenged, but may also turn up novel unexpected functionalities and applications. Advances in peptide medicinal chemistry have eased the way to antiviral PDCs, but challenges remain on the way to therapeutic success. In this paper, we review current antiviral CPP–drug conjugates (antiviral PDCs), with emphasis on the types of CPP and antiviral cargo. We integrate the conjugate and the chemical approaches most often applied to combine both entities. Additionally, we comment on various obstacles faced in the design of antiviral PDCs and on the future outlooks for this class of antiviral therapeutics.

## 1. Introduction

The ongoing SARS-CoV-2 pandemic is a sobering reminder that viruses continue to pose critical challenges to public health worldwide. In addition to SARS-CoV (2002, 2019), the last two decades have witnessed other deadly viral pandemics, such as influenza A (2009), Middle East respiratory syndrome coronavirus (MERS-CoV, 2012), Ebola (2013) and Zika virus (ZIKV, 2016) [1]. In addition to—and because of—their overall health impacts, such outbreaks of emerging and/or re-emerging viral infections disrupt the global economy, often with devastating effects on public welfare. Altogether, their biological, environmental and socio-economic impacts make viruses and virus-related diseases one of the greatest challenges to humankind [2,3].

Dealing with viral disease requires addressing still unsolved issues, such as (i) a limited arsenal of antiviral drugs with often narrow activity (e.g., diverging effects on different virus subtypes) [4], (ii) in vivo delivery hurdles (toxicity, solubility, efficacy, safety, etc.) [5] or (iii) high mutation rates, leading to drug resistance and ensuing therapeutic failure [6]. In over half a century since the launch of the first antiviral drug, idoxuridine, in 1963, only 90 other approvals have been recorded, which have been catalogued into 13 groups for treating just 9 human viral infections [7]. They largely rely on relatively small molecules—e.g., proteases, polymerases or integrase inhibitors. Clearly, development of effective antiviral therapies remains a pressing need, in whose pursuit not only must viruses be targeted, but host cell processes in the virus life cycle as well [8].

Peptides are making steady inroads into diverse therapeutic categories, particularly as anti-infectives. Antimicrobial peptides (AMPs) have proven successful against various microbial infections; 11 AMPs are FDA-approved and 19 are currently under clinical trials [9], mostly targeting bacterial infections. In contrast, the number of peptides studied for antiviral action (AVPs) is much lower, roughly a third of those validated as antibacterial [10]. Even so, given the challenges posed by viral disease, it is no surprise that a number of peptide-inspired endeavors are underway, not only involving AVPs, but more attuned to this work, peptide conjugates with the ability to cross biological barriers and to exert their functions in specific and efficient ways.

The field of peptide-drug conjugates (PDCs) has grown considerably over recent decades, as a regular flow of candidate conjugates have entered clinical trials [11] aimed at cancer, diabetes, Alzheimer’s disease or microbial infections caused by *Escherichia coli, Pseudomonas aeruginosa, Salmonella,* etc. However, as with AVPs, antiviral PDCs are still scarcely represented in drug pipelines. In most of those few instances, the approach involves chemical linkage of an established (small molecule) antiviral to a cell-penetrating peptide (CPP) to achieve efficient delivery at a particular intracellular target.

**Figure 1:**
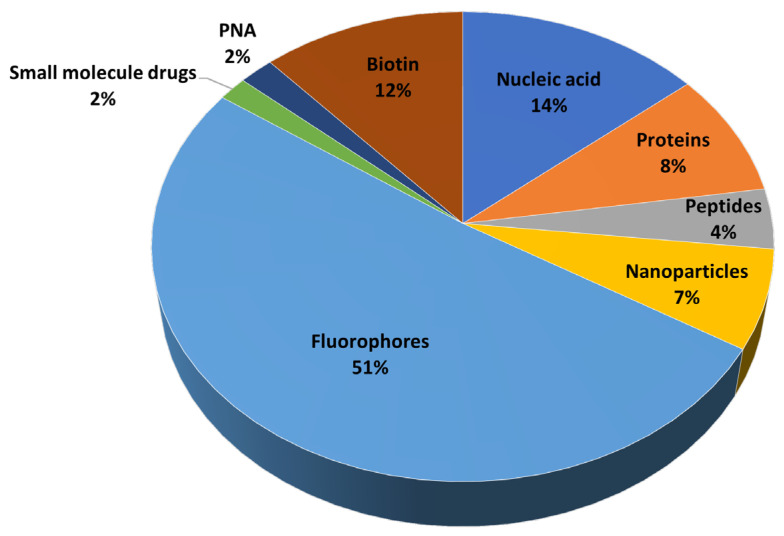
Types of cargoes delivered into cells by CPPs (data from [14]).

CPPs are typically 5–30 amino acid residues in size and structurally diverse but usually cationic. They have the ability to translocate bioactive payloads into living cells [12]. CPPs successfully deliver into cells diverse types of cargoes, often exceeding (~200 times) typical bioavailability size restrictions [13]. As shown in Figure 1 (data taken from [14]), the most frequent cargoes are fluorescent dyes used mainly for diagnostic purposes, but conjugates delivering nucleic acids, proteins, nanoparticles, therapeutic peptides or other payloads of biomedical relevance are also favored.

Since the discovery of the first CPPs (HIV-Tat, penetratin) [15,16], many other sequences have been documented, including synthetic and chimeric ones [17,18,19,20,21,22]. The CPPsite 2.0 database provides detailed updated information on hitherto reported CPPs and their categorization based on various criteria (Table 1).

In this review, we discuss current antiviral cell-penetrating peptide–drug conjugates (antiviral CPPDCs), focusing on the types of CPP and cargo involved, the conjugation chemistries used and their effects on conjugate performance. We close by discussing future perspectives of antiviral PDC application.

## 2. PDCs and Antiviral Cargoes

Drug conjugates are chemotherapeutic agents consisting of drug cargo bound to a carrier (antibody, CPP), either directly or through a linker unit. Most currently available PDCs are designed as new modalities of targeted therapy, with improved efficacy and reduced side effects, against various cancer types [23,24]. There are so far only two FDA-approved PDCs on the market, but several others are at various stages of clinical trials [23,24,25]. He et al. [26] have reviewed the technologies for conjugation of CPPs and small drugs and their outcomes as cancer therapeutics. Indeed, most entries in the comprehensive ConjuPepDB database of PDCs are antitumoral [27]. Even so, PDCs are receiving increasing attention as antimicrobials, as judged by the non-negligible number (224) of ConjuPepDB entries retrieved by the query “antimicrobial”. More importantly, and pertinently to this work, of those 224 PDCs, 118 are also retrieved by the query “antiviral”, 7.2% of total entries in the database [27].

Table 2 lists all antiviral PDCs reported so far, specifying the CPP sequence, antiviral cargo, the conjugation chemistry, the targeted virus and the experimental screen used for validation.

Peptide design in most of the PDCs presented above is mainly based on already-known CPP sequences (Tat, polyArg, transportan, penetratin) derived from natural protein sequences or resulting from de novo design. In other instances, the sequences are derived from a protease specific substrate (Table 2 entry 20) or from viral protein domain(s) (Table 2, entry 1–8, 21), or are a mix of CPPs with these previous categories (Table 2, some examples in entries 29 and 31). Moreover, in some cases (Table 2, entries 11–19), sequences with membrane-permeating characteristics are used as prodrugs/substrate recognition motifs for proteases. Finally, the majority of CPPs in Table 2 are linear, of L-chirality, cationic or amphipathic; to the best of our knowledge, only two reports on antiviral PDCs based on anionic CPPs have appeared [82,83].

The antiviral payloads are mainly small drug molecules or modified oligonucleotides. In the former case, conjugation to a CPP usually increases the concentration of the therapeutic molecule in body fluids due to improved conjugate solubility—a feature critical for in vivo applications. With modified oligonucleotides (PMO or PNA) as antiviral payloads, conjugation is achieved either through an amide bond or by thiol-ene chemistry via a suitable linker. In addition to their wide-spectrum antiviral activity, these conjugates (Table 2) have been also used as diagnostic tools for the detection of viral nucleic acids [84,85].

## 3. PDC Design Considerations

**Figure 2:**
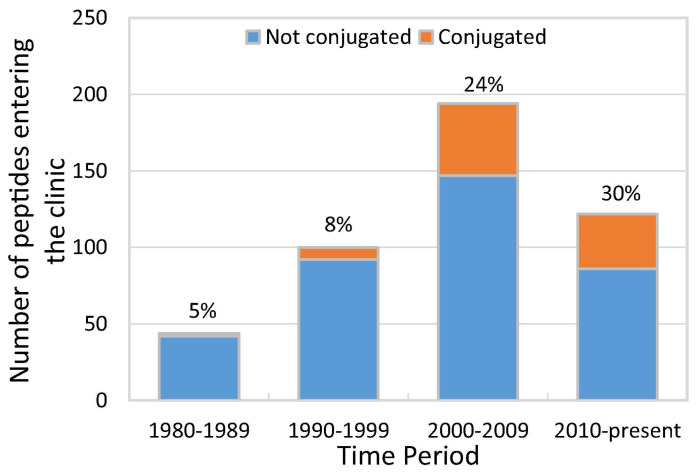
Peptides and peptide conjugates entering clinical trials since 1980s (data from [11]).

While peptide-based medicines remained for years a small fraction of pharmaceutical business—and PDCs an even smaller one—the situation changed in the 1990s (Figure 2) when a number of PDCs entered clinical trials. The upward trend continued to the point where between 2010 and 2018, about 30% of peptides in clinical development were conjugates.

While recent, steady advances in peptide chemistry allow relatively straightforward access to PDCs, there are still issues to be addressed in the quest for pharmaceutical success, particularly the following:

(i) A robust enough biological rationale endorsing the combination of the two (peptide + antiviral) or three (peptide + antiviral drug + linker) components of the conjugate is desirable.
(ii) A CPP moiety should be chosen that warrants tissue-specific delivery and hence reduces off-target adverse effects. This ideal scenario is very often ignored, as can be seen in Table 2, where typical CPP sequences with broad spectra of membrane permeability are the ones used in the design of the PDCs.
(iii) Antiviral cargoes active at concentrations commensurate with those of the CPP are desirable. Peptides can act at rather low (e.g., nM) concentrations not met by antiviral molecules. These, in turn, can bind plasma proteins (e.g., albumin) with high affinities that can significantly alter pharmacokinetics and/or pharmacodynamics of the conjugate. Those issues should ideally be considered and harmonized.
(iv) For linker-containing conjugates, the linker should if possible be chosen while bearing in mind factors such as the desirable circulation time for the conjugate to reach its target or the specific location where the drug needs to be released.
(v) Another important consideration is the position where the payload is placed. While the N-terminus of the CPP—elongated or not via an intervening spacer unit—is rather usual, alternative approaches, e.g., by way of an extra residue (often Lys or Cys) at either (N- or C-) end of the proper CPP sequence are also favored. Recent work has shown that whichever of these attachment modes is used can have a significant impact on conjugate performance [82,83].
(vi) The conjugate end-product should ideally be non-cytotoxic, non-immunogenic and have minimal interference (hence adverse reactions) with other drugs in multi-therapy schedules.
(vii) Finally, uptake mechanisms ensuring successful release of the antiviral drug from the PDC need to be elucidated. CPP internalization (with or without cargo) is a complex process with multiple factors (positive charge, amphipathicity, folding ability, cargo structure or cell internalization, through active (energy-dependent) or passive (energy-independent) penetration pathways) influencing the peptide–membrane interaction, which is crucial for successful outcomes [86,87,88,89,90].

Despite the complexities of the design process and the high number of factors influencing conjugate performance, by carefully considering the above-listed aspects, it is possible to produce effective antiviral PDCs. A suitable algorithm guiding conjugate design based on activity, serum stability and penetration capability would be desirable to speed up the development process. While algorithms derived from random peptide library design [91], machine learning-based predictive models [92] and natural sequence scanning [93] have been proposed for predicting peptide bioactivity, unfortunately, no such tool is yet available for peptide–antiviral-drug conjugate activity. Future studies should also focus on PDCs with various therapeutic profiles for further development in stability and performance.

## 4. Conjugation Chemistry

**Figure 3:**
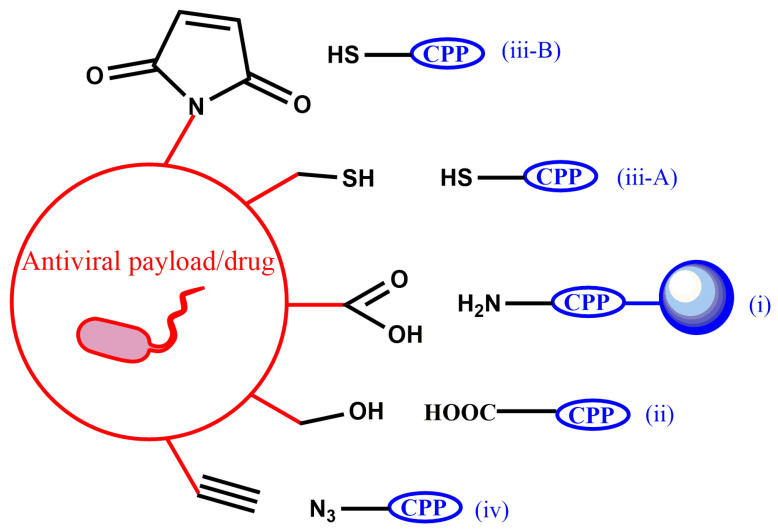
Conjugation chemistries applied in the synthesis of antiviral PDCs. Labels (i, ii, iii-A, iii-B and iv) as described in the text above. In amide bond formation (i), conjugation is performed while the CPP is anchored on the solid support. In thioether bond formation (iii-B), a thiol-maleimide reaction is depicted. In (iv), copper (I)-catalyzed alkyne-azide cycloaddition is shown as a representative click reaction.

Although joining two different chemical entities into a single molecule while preserving their physico-chemical properties can be challenging, synthetic strategies to obtain antiviral PDCs, either in solution or on solid supports, are relatively well established. The latter approach exploits the advantages of solid-phase peptide synthesis plus the chemoselectivity provided by specific amino-acid side chains. In all examples in Table 2, conjugation between CPP and antiviral cargo is achieved through a covalent bond whose formation has as main requirement that the CPP, the cargo and the resulting conjugate are stable throughout the synthesis and purification steps. In practice, five types of conjugation chemistries are favored: (i) amide bond formation, (ii) ester bond formation, (iii) thiol-based chemistries that comprise disulfide, (iii-A) thioether bond formation (including thiol-ene coupling, iii-B) and (iv) click reaction (Figure 3).

Usually, the methods based on amide bond formation employ peptide *N*-terminal conjugation or lysine-residue-selective activation and coupling while the peptide is still anchored to the solid support. In the other three coupling strategies, the activation and corresponding chemical bond formation are predominantly performed in solution.

### 4.1. Conjugation Based on Amide Bond Formation

Amide bond formation, a straightforward and versatile way to attach a drug molecule to a peptide [11,94,95,96,97], is a preferred conjugation option whenever possible, affording substantial stability towards acid or basic media, temperature, etc. Their chemical stability is, however, counterbalanced by their often rather short lifespans in biological fluids and cell compartments (e.g., lysosomes) owing to peptide proteolytic susceptibility [98,99,100,101]. To mitigate this limitation, integration into various platforms (polypeptide carriers, liposomes, nanocarriers, etc.) has been proposed as providing steric protection and increased in vitro or in vivo lifetimes [101,102,103]. A few PDCs with the antiviral cargo directly attached to the CPP through an amide bond have so far been described [29,32,34]. For example, Wang et al. [29] and Nitsche et al. [34] reported conjugation through the CPP *N*-terminus, and Diez-Torrubia et al. [32] attached the antiviral payload by direct reaction with the *C*-terminal carboxyl group. This latter study also showed that amide-based conjugates acting as acyclovir carriers provided faster release of the antiviral compared to ester-linked ones. For its part, in the study of Nitsche et al. describing a new generation of PDCs acting as dengue virus protease inhibitors, the position at which the 5-arylidenerhodamine or 5-thiazolidinedione cargo was linked by amide bond to the CPP was consequential [34,104], and a structural change in the payload (e.g., S by O exchange; thiazolidinone vs. rhodamine, respectively) likewise altered the interaction profile of the conjugate [34].

In a study by Moulton et al., it was observed that the CPP used is quite relevant for successful delivery of PMO conjugates [105]. These findings were in tune with those of Abes et al., who showed that a particular CPP (R-Ahx-R)_4_ was much superior in delivering PMOs to the nucleus [106]. In recent reports on antiviral-peptide–porphyrin conjugates, Mendonça et al. and Todorovski et al. evaluated various amide-based conjugation schemes (*N*-terminal, C-terminal via an extra Lys, with or without an intervening PEG-based linker) and showed that the linker between the CPP and the cargo can influence aspects such as cell penetration, toxicity, antiviral activity [82,83]. Finally, it is worth mentioning here that in all currently described PDCs with PMO or PNA as antiviral cargo, a suitable linker is present between both moieties (Table 2, entries 22–30).

### 4.2. Conjugation Based on Ester Bond Formation

Although, in comparison to other covalent linkages, ester bonds do not provide high chemical or plasma stability, they are nonetheless widely used for conjugating drugs to peptides given their relatively simple synthesis and their well-characterized cleavage mechanisms, either by esterases or under acidic conditions [26]. Ester bond-linked antiviral PDCs have already been mentioned above as anti-herpes agents [32], and Liotard et al. reported higher chemical stability (e.g., hydrolysis resistance) of HIV-targeting ester-conjugated prodrugs compared to phosphoramidate-based analogs [33]. In most cases, the antiviral activity of these conjugates is correlated to their hydrolysis rates. Prodrug conjugates with Boc or Fmoc protection at the CPP N-terminus were generally less stable than those with Z- or Qnc protecting groups [33]. Despite the authors’ conclusion that the data obtained do not validate their initial hypothesis for designing HIV RT prodrugs, the work offers helpful insights on the importance of the synthetic chemistry used, the position where the conjugation is performed and the overall stability.

### 4.3. Conjugation Based on Thiol Chemistry

The ability of thiols to act as nucleophiles in a variety of reactions with nearly quantitative yields has long made them attractive groups for conjugation. Among the various reaction types reported (thiol-halogen, thiol-maleimide, thiol-ene, etc.) [107], only antiviral PDCs based on disulfide, thioether (including thiol-maleimide) or thiol-halogen linkages have been reported so far (Table 2).

Disulfide formation is most often achieved in a directed, efficient manner by reaction of a Cys residue in the peptide with an activated (electrophilic) thiol group in the antiviral drug. Intracellular release of disulfide-linked payloads is glutathione-trigged; in contrast, for thioether or thiol-maleimide PDCs, there are no specific enzymes able to release the payload, although decomposition by oxidation or β-elimination has been reported for long plasma exposure [108,109]. A large number of PMO- and PNA-based antiviral PDCs are based on either of these approaches (Table 2, entries 23–30), following Moulton et al. [100]. These reports also show that linker chemistries used for antisense PMO delivery did not significantly affect activity but did influence uptake and intracellular distribution of the conjugate [105]. Turner et al. compared the antiviral activities of PNA-CPP conjugates formed by either amide or disulfide linkages and demonstrated better performance for the latter ones [67]. Additionally, Kumar et al. reported the systemic delivery of antiviral siRNAs to T cells by way of a single-chain antibody conjugated to a poly-Arg CPP through a disulfide linkage [76]. For their part, Meng et al. (Table 2, entry 31) were able to successfully conjugate siRNA to HIV-1 TAT_47-57_, using thiol-maleimide coupling, and to show that the corresponding conjugate could effectively block hepatitis C virus replication in the cells [75]. Another report based on thiol-maleimide conjugation was by Zeng et al. (Table 2, entry 30), where a PNA conjugate exerted considerable inhibitory effects against hepatitis B virus in vitro and in vivo [72]. To the best of our knowledge, these two reports, along with that by Moulton et al., are the only examples where thiol-maleimide chemistry is used to produce the antiviral agent. However, further research in this area is needed to clarify whether and how cleavable or stable linkages influence antiviral activity by their stability (or lack thereof) in specific microenvironments.

### 4.4. Conjugation Based on Click Chemistry

The recently awarded Nobel prize in Chemistry for the “development of click chemistry and bioorthogonal chemistry” testifies about the importance of efficient reactions that allow the formation of functional bioconjugates. In particular, the Cu(I)-catalyzed azide-alkyne cycloaddition (CuAAC) takes place under mild conditions and does not interfere with the Cys or Lys residues that, as discussed above, are frequent players in conjugation chemistries. The triazole ring resulting from CuAAC is stable against enzymatic degradation, reduction, hydrolysis and oxidation. Somehow, surprisingly, examples of antiviral conjugates made by this approach are mainly limited to the paper by Liang et al., where PDCs able to inhibit HIV-1 mediated cell fusion and infection are described [28]. Two of the described conjugates (those with indole and Gls, Table 2, entries 1 and 2, correspondingly) showed 6-fold improved inhibitory activity compared to the clinically used fusion inhibitor. Additionally, Zhou et al. compared the ability of various antiviral PDCs, obtained by either click chemistry and thiol-chloroacetyl reaction, to activate human RNase. The latter ones were 3-fold more potent [30], which in turn led to more efficient rRNA cleavage.

## 5. Future Perspectives

PDCs combining a CPP and a relatively small drug rely with few exceptions on a covalent bond between both moieties to create a single entity that achieves efficient cell penetration and antiviral activity. Despite the promising outlook, the modest success rate indicates that important drawbacks, more of biological than chemical nature, still need to be overcome.

(1) Selectivity remains a substantial challenge in that it is rare to find extracellular targets (i.e., receptors recognized by the CPP) expressed at a distinct tissue. Thus, antiviral PDCs may access any cell or tissue where a particular receptor is expressed, with the risk of causing off-target toxicity in healthy cells. To address this issue, three solutions, well researched and applied in the anticancer CPP-drug conjugate field, can be advanced: (i) cell- and tissue-specifically designed CPPs, (ii) conjugation of existing CPPs to suitable targeting entities and (iii) modulation of CPP uptake by a stimulus-sensitive signal. In the first strategy, the target-specific CPPs are usually isolated through phage-display [22,110,111,112]. This technology, awarded a Nobel Prize in Chemistry in 2018, allows high-affinity target-binding peptides to be selected from a complex pool of billions of peptides displayed on a phage and has become one of the most common methods for the identification of specific peptide ligands to virtually any target [112]. In the second approach, improved targeting by the PDC is typically achieved by endowing the conjugate with an additional, tissue-specific homing unit such as folic acid, transferrin and antibodies [113,114,115]. In the third strategy, the most used in targeted delivery of anticancer PDCs, CPP uptake is impaired until a specific stimulus (pH, temperature, light, proteolysis) triggers cell membrane penetration [22]. pH-responsive anticancer PDCs are most favored, due to the significant pH differences between cancer and healthy cells [116]. Unfortunately, virus-infected cells do not display such a pH imbalance; therefore, a next-best approach would be to develop antiviral PDCs that can be activated on demand by virus-specific enzymes.

(2) Another challenge to obtaining stable conjugates is proteolytic instability [117,118]. As CPPs (made up of L-amino acids) are prone to degradation in biological fluids, approaches such as switching to all- or partial-D configuration, or modifications to specific residues or the backbone, or using cyclic CPPs, have shown that this obstacle can be successfully addressed. Additionally, the linkage between the CPP and the drug is another vulnerable element easily recognized and cleaved (ester and amide-bond based linkages) by certain proteases in biological fluids. This is not so with thiol-maleimide and triazole-based junctions, which are protease-stable and should be preferred when a long circulation and stability of the PDC is required, or when the PDC unit has better antiviral activity than the drug alone. In contrast, if a well-controlled intracellular release of the conjugated drug is required, disulfide and ester-based conjugation chemistries should be employed. In those instances when an intervening linker unit is used to connect CPP and cargo, additional issues may be considered. For example, a particular type of CPP–cargo junction, receiving significant attention over recent decades within the PDC field [119], is that of so-called self-immolative linkers (SILs), designed to degrade spontaneously in response to specific stimuli [120]. Unfortunately, to the best of our knowledge, no antiviral PDCs have been reported so far using this technology for selective and targeted drug delivery within the infected cells. The approach is mainly used in cancer therapeutics, but any progress in identifying specific viral enzymes/proteins that can recognize and cleave certain SILs would become a real breakthrough within the field of antiviral PDCs.

(3) A further challenge has to do with our limited knowledge of intracellular biochemistry—i.e., after endocytosis of a conjugate, it is not yet clear how the internalized molecule avoids lysosomal degradation—a step critical for successful release into the cytosol. Furthermore, even when endosomal escape and intracellular delivery are successful, the mechanism of final transport to the preferred intracellular destination (mitochondria, nucleus, other sites) is largely unknown and constitutes an active field of study. Moreover, once the desired biological effect is achieved, issues such as its switch-off and the related question of possible reverse extracellular transport still stand open. On a related tune, unintended PDC effects on tissues/cells—avoided at the entry but unknown at the exit phase—need to be explored.

(4) An equally open challenge, as novel PDCs continue to be developed, is the fine-tuning of activities between the CPP component and its small molecule cargo, to avoid potency disparities.

(5) In vivo trials of PDCs into the pipeline must include not only mandatory toxicity and immunogenicity tests, but also, for antiviral PDCs, ensure that viral inactivation does not entail any deleterious effects on the host cell’s machinery [121].

(6) Finally, given the high genetic and antigenic heterogeneity of viruses, multivalency screens on different strains/isolates must be performed to avoid that conjugates with promise against one particular virus are inefficient against other types or subtypes.

The speed at which these challenges are addressed will determine, to a great extent, the future of antiviral therapies using PDCs and their ability to compete/replace existing unmodified drug formulations.

## Figures and Tables

**Table 1:** Reported CPPs and categories (from CPPsite 2.0, [14]).

		Total Numbers	Percentage (%) ^a^
Sequence type	Linear	1753	94.5
	Cyclic	102	5.5
Peptide class	Cationic	714	38.5
	Amphipathic	391	21.1
Origin	Protein	774	41.7
	Synthetic	1017	54.8
	Chimeric	64	3.5
Chirality	L	1564	84.3
	D	63	3.4
	Mixed	32	1.7
	Modified	110	5.9
Length	Up to 5 AA	60	3.2
	6–10 AA	384	20.7
	11–15 AA	550	29.6
	16–20 AA	446	24.1
	21–30 AA	320	17.3
	>30 AA	95	5.1

^a^ Relative to the total number of peptides (1855) in the database.

**Table 2:** Antiviral PDCs ^a^ reported.

Entry	Antiviral Cargo	CPP	Conjugation Chemistry	Targeted Virus	Experimental System	Literature
1	Indole	βAla-EYAARIEALIRAAQEQQEKNEAALRE	Click chemistry	HIV-1	Cell culture (HL2/3 and MT-2 cells)	[28]
2	*N*-carboxyphenylpyrrole derivative (Gls)					
3	Indole	βAla-EYAARIEALIRAAQEQQKKNEE				
4	*N*-carboxyphenylpyrrole derivative (Gls)					
5	Indole	βAla-EYAARIEALIRAAQEQQKK				
6	*N*-carboxyphenylpyrrole derivative (Gls)					
7	Carboxymethyl derivative of *N*-(3-carboxy-4-hydroxyphenyl)-2,5-dimethylpyrrole (Aoc)	βAla-NNYTSLIHSLIEESQNQQEKNEQELL	Amide bond formation	HIV-1	Cell culture (HL2/3 and MT-2 cells)	[29]
8	Carboxymethyl derivative of *N*-(4-carboxy-3-hydroxyphenyl)-2,5-dimethylpyrrole (Noc)					
9	2-5A 2′ 5′-phosphodiester linker oligoadenylate	GGRRKKRRQRRR (HIV-Tat)	Click chemistry	HIV	Cell culture (HeLa M cells)	[30]
10	2-5A 2′ 5′-phosphodiester linker oligoadenylate	CGGRKKRRQRRR (HIV-Tat)	Thiol-chloroacetyl ligation			
11	*N*-3 aminopropyl TSAO-T	VAVP	Amide bond formation	HIV-1	Cell culture (Human T lymphocytic CEM and MT-4 cells)	[31]
12		VAVA				
13		KPDP				
14	Acyclovir	VPVP	Amide bond formation	HSV-1, HSV-2	Cell culture (HEL cells)	[32]
15		VPV	Ester bond formation			
16	Zidovudine (AZT)	Boc-FP; Boc-NFP; Boc-FPI; Boc-NFPI; Fmoc-FP; Fmoc-NFP; Fmoc-FPI; Fmoc-NFPI; Z-FP; Z-NFP; Z-FPI; Z-NFPI; Qnc-FP; Qnc-NFP; Qnc-FPI; Qnc-NFPI	Ester bond formation	HIV-1	Cell culture (CEM-SS TK^+^, CEM-SS TK^-^ and MT-4 cells)	[33]
17	Zidovudine monophosphate (AZT-MT)	FP-OMe; FPI-OMe; NFP-OMe; NFPI-OMe; AFP-OMe; AFPI-OMe; ANFP-OMe; ANFPI-OMe	Phosphoramidate bond formation			
18	Rhodanine ^b^	Arg-Lys-Nle	Amide bond formation	Dengue virus, West Nile fever virus	Cell culture (Huh-7 cells)	[34]
19	Thiazolidinedione ^a^					
20	GRL0617 (C_20_H_20_N_2_O)	ECLRGM (cyclic)	Amide bond formation	SARS-CoV-2	Cell culture (Human kidney cells 293T; Human lung adenocarcinoma A549 cells; HCT116 cells)	[35]
		EMLRGC (cyclic)				
21	25-Hydroxycholesterol (25-HC)	SLDQINVTFLDLEYEMKKLEEAIKKLEESYIDLKELGSGSG	Amide bond formation through linker	SARS-CoV-2	Human kidney 293T cells; Huh-7 cells; RD cells; Caco2 cells	[36,37]
	Palmitic acid (C16)					
22	PMO	(RAhx ^c^ R)_4_	Amide bond formation through linker	SARS-CoV-2	Vero-E6 cells	[38]
23	PMO	(RAhxR)_4_-Ahx-βAla	Amide bond formation through linker; thioether bond formation through linker;	West Nile fever virus, Japanese encephalitis virus, St. Louis encephalitis virus, Coxsackievirus B2, Coxsackievirus B3, poliovirus 1, human rhinovirus 14, mouse hepatitis virus, Venezuelan equine encephalitis virus, respiratory syncytial virus, measles virus, influenza A virus, Kaposi’s sarcoma-associated herpesvirus, herpesvirus type 1	Cell culture (KSHV-infected BC-1 and BCBL-1 cells; MDCK cells; Vero or Vero/hSLAM cells; HeLa and HL-1 cells; BHK-21 cells), in vivo mouse infection model	[39,40,41,42,43,44,45,46,47,48,49,50,51]
24	PMO	RRRRRFFRRRRC; RRRRRRRRRFFC; (RAhxR)_4_-Ahx-βAla	Amide bond formation through linker; thioether bond formation through linker	Dengue virus	Cell culture (Vero and BHK-21 cells), in vivo mouse infection model	[52,53,54]
25	PMO	RRRRRFFRRRRC; RRRRRRRRRFFC	Thioether bond formation through linker;	SARS-CoV1	Cell culture (Vero-E6 cells)	[55]
26	PMO	RRRRRRRRRFFC	Thioether bond formation through linker	Equine arteritis virus, foot-and-mouth disease virus, poliovirus 1, human rhinovirus 14, coxsackievirus B2, Mouse hepatitis virus, Sindbis virus	Cell culture (BHK-21 and Vero cells; DBT cells; HeLa cells; Vero-E6 cells)	[41,44,56,57,58]
27	PMO	RRRRRFFRRRRC	Thioether bond formation through linker	Influenza A virus, porcine reproductive and respiratory syndrome virus, Kaposi򲀙s sarcoma-associated herpesvirus	Cell culture (ATCC CRL11171 cell line; BC-1 and BCBL-1 cells; MDCK cells)	[47,50,59,60]
28	PMO	RRRRRRRRRFFC; (RAhxR)_4_-Ahx-βAla; (RβAla)_8_βAla; (RAhx)_n=2-8_βAla	Amide bond formation through linker; thioether bond formation through linker	Ebola virus	Cell culture (Vero and Vero-E6 cells) in vivo mouse infection model	[61,62]
29	PNA	CGWTLNSAGYLLGKINLKALAALAKKIL; (Npys) ^d^ GWTLNSAGYLLGKINLKALAALAKKIL; RQIKIWFQNRRMKWKK; GRKKRRQRRRPPQ; GWYLNSAGYLLGK(Cys)INLKALAALAKKIL; AGYLLGK(Cys)INLKALAALAKKIL; GWYLNSAGYLLGK(Cys)INLKALAAL; GRKKRRQRRRP; GWTLNSAGYLLGKINLKALAALAKKIL; GWYLNSAGYLLGKINLKALAALAKKIL; PKKKRKV; GRKKRRQRRRPC; RQIKIWFQNRRMKWKKGGC; RRRRRRRRRFFC; RRRRRRRQIKIWFQNRRMKWKKGGC	Disulfide bridge formation; amide bond formation through linker	HIV-1	Cell culture (HeLa cells; 293T cells; CEM CD4^+^ cells; Jurkat T-cell lymphocites; Vero and Vero E6 cells). in vivo mouse infection model	[63,64,65,66,67,68,69,70]
30	PNA	GRKKRRQRRRPPQ; GRKKRRQRRRPPC; YGRRRRRRRRR; RKKRRQRRR	Amide bond formation through linker; thiol-maleimide bond formation	Japanese encephalitis virus, hepatitis B virus, hepatitis C virus, SARS-CoV	Cell culture (Huh7 cells; Vero and BHK-21 cells; HepG2.2.1.5, HepG2 and L-02 cells), in vivo mouse infection model	[71,72,73,74]
31	siRNA	CYGRKKRRQRRR; RRRRRRRRR; KETWWETWWTEWSQPGRKKRRQRRR; GWTLNSAGYLLGKINLKALAALAKKILrrrrrrrrr ^e^	Disulfide bridge formation; thiol-maleimide bond formation; non-covalent complex formation	Hepatitis C virus, HIV-1 influenza virus	Cell culture (Huh7 cells; MDCK and A549 cells; MDM cells), in vivo mouse infection model	[75,76,77,78]
32	Protein	RRRRRRRRR; YGRKKRRQRRR	Cell expression	Human papilloma virus type 18, hepatitis B virus, mucosal influenza	Cell culture (MDCK cells; Huh7 and HepG2.2.1.5; human cell line 293H), in vivo mouse infection model	[79,80,81]
33	Porphyrin	AGILKRW AGILKRWK VQQLTKRFSL VQQLTKRFSLK SGTQEEY SGTQEEYK	Amide bond formation	HIV-1 Zika virus	Cell culture (Vero and TZM-bl cells)	[82,83]

^a^ We have pondered whether the acronym CPPDC would describe the conjugates more accurately than PDC, since to our best knowledge all reported PDCs contain a cell-penetrating peptide (CPP) motif, but have finally decided for the more widely accepted PDC acronym. ^b^ Twenty-six different rhodamine and thiazolidinedione substituents were evaluated. ^c^ Ahx stands for 6-aminohexanoic acid. ^d^ Npys stands for 3-nitro-2-pyridinesulfenyl. ^e^ Lower case denotes D-amino acid residues.

## Data Availability

Not applicable.
